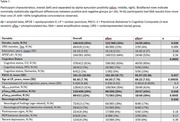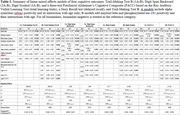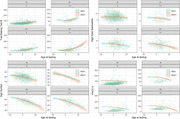# Seed amplification assay‐determined alpha‐synuclein associates with Alzheimer’s pathology, clinical symptoms, and cognitive change

**DOI:** 10.1002/alz.089741

**Published:** 2025-01-09

**Authors:** Erin M. Jonaitis, Karen R MacLeod, Lamoureux Jennifer, Beckie Jeffers, Rachel L Studer, John Middleton, Rachael E Wilson, Nathaniel A. Chin, Bruce P Hermann, Sean McEvoy, Henrik Zetterberg, Sterling C. Johnson, Russ Lebovitz, Rebecca E. Langhough

**Affiliations:** ^1^ Wisconsin Alzheimer’s Institute, University of Wisconsin‐Madison School of Medicine and Public Health, Madison, WI USA; ^2^ Wisconsin Alzheimer's Disease Research Center, Madison, WI USA; ^3^ Amprion, San Diego, CA USA; ^4^ University of Wisconsin‐Madison, Madison, WI USA; ^5^ Department of Medicine, University of Wisconsin‐Madison School of Medicine and Public Health, Madison, WI USA; ^6^ University of Wisconsin‐Madison, School of Medicine and Public Health, Madison, WI USA; ^7^ Amprion, San Francisco, CA USA; ^8^ Wisconsin Alzheimer’s Disease Research Center, University of Wisconsin School of Medicine and Public Health, Madison, WI USA; ^9^ Institute of Neuroscience and Physiology, Sahlgrenska Academy at the University of Gothenburg, Mölndal, Gothenburg Sweden; ^10^ Dementia Research Centre, Department of Neurodegenerative Disease, UCL Queen Square Institute of Neurology, University College London, London, United Kingdom, London United Kingdom; ^11^ Department of Psychiatry and Neurochemistry, Institute of Neuroscience and Physiology, The Sahlgrenska Academy at the University of Gothenburg, Mölndal Sweden; ^12^ Wisconsin Alzheimer's Disease Research Center, University of Wisconsin School of Medicine and Public Health, Madison, WI USA; ^13^ Wisconsin’s Alzheimer’s Institute, School of Medicine and Public Health (SMPH), University of Wisconsin‐Madison, Madison, WI USA

## Abstract

**Background:**

The high prevalence of mixed‐pathology dementias suggests that multi‐drug treatments may improve clinical outcomes; thus, in‐vivo biomarkers for co‐pathologies are needed. We investigated a novel assay for detecting seeds of misfolded alpha synuclein (αSyn) and explored its relationship to outcomes including Alzheimer’s disease (AD) biomarkers, clinical features, and cognitive trajectories, in two community‐based cohorts enriched for AD risk.

**Method:**

Cerebrospinal fluid (CSF) obtained from participants in the Wisconsin Registry for Alzheimer’s Prevention and the Wisconsin Alzheimer’s Disease Research Center (N=418 participants; 515 LPs; Table 1) was assayed using a clinically validated, qualitative Syn seed amplification assay (SAA; Amprion). Co‐occurrence of αSyn pathology (αSyn‐SAA+), and AD pathology (CSF amyloid, Aβ_42/40_ and pTau_181_/Aβ_42_, and tau, pTau_181_, positivity), and clinical symptoms (neurological finding; Parkinsonian signs; etc) were examined with chi‐square tests. Cognitive trajectories were modeled retrospectively using separate mixed effects models of Trail‐Making Test B, Backward Digit Span, Digit Symbol Substitution, and a three‐test Preclinical Alzheimer’s Cognitive Composite (PACC‐3). Models included subject‐level random intercepts and age slopes and standard covariates (sex, education, and practice with the battery). For each outcome, we examined whether αSyn‐SAA+ modified age trajectories (centered; linear and quadratic terms; models 1A‐4A). In models 1B‐4B, we further examined effects of αSyn‐SAA+ after adjusting for age× and age× interactions. Nonsignificant quadratic interaction terms were removed (p>0.1).

**Result:**

αSyn‐SAA+ co‐occurred with binary pTau_181_/Aβ_42_ (p=0.048) and pTau_181_ (p=0.0045), but not Aβ_42/40_ (p=0.14). Among pTau_181_/Aβ_42_+ participants, 20/119 (17%) were also αSyn SAA+, compared to 27/287 (9%) pTau_181_/Aβ_42_‐ participants. For pTau_181_+ and pTau_181_‐, the proportions were 18/89 (20%) and 30/322 (9%), respectively. αSyn‐SAA+ participants had greater cognitive impairment (p=0.00050) and additional neurological findings (p=0.026), but did not differ (p>=.17) on Parkinsonian signs, REM behavior disorder, or depression. αSyn‐SAA+ participants declined faster in Digit Symbol performance after accounting for Alzheimer’s pathology (model 3B: = ‐0.20, p=0.045; Table 2; Figure 1).

**Conclusion:**

Results suggest significant co‐pathology in an AD‐risk enriched cohort. Relationships with cognitive impairment provide evidence for the clinical utility of αSyn SAA in this population. Steeper age‐related decreases in Digit Symbol performance among αSyn SAA+ individuals suggest possible association with preclinical cognitive change.